# Exploring the Potential of Statistical Modeling to Retrieve the Cloud Base Height from Geostationary Satellites: Applications to the ABI Sensor on Board of the GOES-R Satellite Series

**DOI:** 10.3390/rs13030375

**Published:** 2021-01-22

**Authors:** Pedro A. Jiménez, Tyler McCandless

**Affiliations:** 1 National Center for Atmospheric Research, Boulder, CO 80301, USA; 2 E Source, Boulder, CO 80301, USA;

**Keywords:** cloud base height, ABI, GOES-16, METAR, random forest, machine learning

## Abstract

Although cloud base height is a relevant variable for many applications, including aviation, it is not routinely monitored by current geostationary satellites. This is probably a consequence of the difficulty of providing reliable estimations of the cloud base height from visible and infrared radiances from current imagers. We hypothesize that existing algorithms suffer from the accumulation of errors from upstream retrievals necessary to estimate the cloud base height, and that this hampers higher predictability in the retrievals to be achieved. To test this hypothesis, we trained a statistical model based on the random forest algorithm to retrieve the cloud base height, using as predictors the radiances from Geostationary Operational Environmental Satellites (GOES-16) and variables from a numerical weather prediction model. The predictand data consisted of cloud base height observations recorded at meteorological aerodrome report (METAR) stations over an extended region covering the contiguous USA. Our results indicate the potential of the proposed methodology. In particular, the performance of the cloud base height retrievals appears to be superior to the state-of-the-science algorithms, which suffer from the accumulation of errors from upstream retrievals. We also find a direct relationship between the errors and the mean cloud base height predicted over the region, which allowed us to obtain estimations of both the cloud base height and its error.

## 1. Introduction

Cloud base height (CBH) is an important meteorological variable in many applications which routinely require accurate monitoring and prediction. For example, accurate CBH monitoring is relevant for aviation in order to avoid the hazards associated with low visibility as a result of flying through clouds [1]. Accurate CBH monitoring is also relevant for its prediction. Numerical weather prediction (NWP) models require the initialization of the atmospheric state which includes the cloud field. Thus, having the correct CBH at the initial time is the first step towards modeling its evolution. Indeed, a proper cloud initialization not only contributes to better cloud forecasts but also to enhancing the general performance of forecasts. For instance, a correct representation of the CBH is necessary to properly represent the surface energy balance. This is a result of the strong impact that the location of the CBH exerts on the net radiative balance at the surface through both modulations of the downward longwave radiation (lower CBHs are typically warmer) and the downward shortwave radiation (thicker clouds attenuate more). This last aspect is relevant for solar energy applications, since the shortwave irradiance at the surface determines power production [2]. Accurate cloud prediction is also important for air quality forecasting, especially for ozone forecasting, because clouds significantly modulate ultraviolet radiation—the relevant portion of the spectrum in tropospheric photochemical ozone formation [3]. Thus, there is a widespread need for accurate CBH prediction and monitoring.

The monitoring of atmospheric variables is a key component of geostationary satellites. These satellites provide high spatio-temporal samplings and low latencies. For instance, the Advanced Baseline Imager (ABI, [4]) on board of the Geostationary Operational Environmental Satellite-R series (GOES-R) provides images at 0.5, 1 or 2 km, depending on the observation band over the Contiguous U.S. (CONUS) every 5 min and over the full disk every 10 min. Additionally, the ABI sensor has a similar spectral coverage to polar-orbiting sensors such as the Moderate Resolution Imaging Spectroradiometer (MODIS) or the Visible Infrared Imaging Radiometer Suite (VIIRS), which should contribute to the enhancement of retrieval performance with respect to older GOES satellite series and/or to the retrieval of additional meteorological variables. However, the GOES-R satellites do not provide CBH retrievals, probably because the retrieval of CBH from passive imaging radiometers is challenging.

The challenge in retrieving CBH lies in the spectral bands observed. Imagers sampling visible and infrared (IR) radiances only provide information of the cloud-top properties and vertically integrated cloud content [5], which makes it difficult to retrieve the CBH. As a result, some of the CBH retrieval methods make use of additional observations. Some methods use ground observations (e.g., [6,7]), whereas other methods use data from active sensors on board of other satellites [8–11]. The methods that use ground observations present limitations over regions with scarce samples such as the ocean. Similarly, the limited sampling of active sensors on board of circumpolar satellites makes it difficult to apply these kinds of retrievals to monitor the CBH from geostationary satellites.

Methodologies that rely only on data from passive sensors are better suited for environmental monitoring. Some methodologies target the CBH retrievals for specific type of clouds (e.g., Meerkotter and Zinner 2007). More general-purpose methodologies combine retrievals of cloud-top properties with retrievals of the cloud geometric thickness (CGT) to estimate the CBH. For instance, the cloud top pressure has been combined with CGT estimations [12,13]. This methodology has the largest uncertainties for optically thick clouds. Analogously, cloud top height (CTH) retrievals can be combined with CGT retrievals to estimate the CBH as the residual [14,15]. This algorithm has been implemented to produce operational CBH retrievals from the VIIRS sensor on board of the Suomi National Polar-Orbiting Partnership (S-NPP) satellite [16]. The comparison of CBH retrievals from S-NPP against CBH retrievals from the active sensor on board of CloudSat presents errors exceeding the 2 km error requirement for the mission and a root mean square error (RMSE) of 3.7 km [17].

We hypothesize that a large portion of the CBH errors is a consequence of the accumulation of errors from upstream retrievals. For example, if the CBH is calculated as the residual of CTH and CGT, errors in both of these retrievals will add up. In addition, it has been shown that the S-NPP retrieval algorithm is impacted by upstream retrievals, primarily the CTH error [17]. The CGT estimation is also subjected to an accumulation of errors. For example, it has been shown [14] that a 10% error in both the cloud effective radius and cloud optical thickness produces errors of about 20% in the CGT estimation. The error further increases with errors in the liquid water content, which can increase the previous errors by 100%.

Considering the adverse effects due to the accumulation of errors, one may wonder if CBH could be retrieved directly from the observed radiances. The CBH retrievals are ultimately based on the passive radiances and ancillary data such as predictions from numerical weather prediction (NWP) models. This is the information used to retrieve the cloud top properties and the CGT required to estimate the CBH. Thus, it is plausible to hypothesize that an accumulation of errors can be avoided using statistical models to directly estimate the CBH from the observed radiances and ancillary data.

The hypothesis is partially supported by an enhanced version of the S-NPP CBH algorithm. The S-NPP algorithm [16] shows superior performance when statistical modeling is incorporated to reduce the dependency on certain upstream retrievals [5]. The algorithm imposes a couple of linear regressions to estimate the CGT as a function of the cloud water path (CWP), and the regression coefficients are a function of the CTH. This statistical modeling avoids dependencies on the cloud phase/type retrievals. The algorithm is being implemented to retrieve the CBH from the VIIRS sensor on board of the NOAA-20 platform. In spite of the progress, the algorithm is still sensitive to errors in the CTH retrievals and errors in the CGT estimations arising from other upstream retrievals. Removing dependencies on more upstream retrievals, and thus the accumulation of errors, may further improve the CBH retrieval.

In this work, we explore for the first time the potential of statistical modeling to directly retrieve the CBH from the radiances from geostationary satellites. The originality of our approach is motivated by the desire of avoiding the accumulation of errors that affects some of the state-of-the-science CBH retrieval algorithms. Our approach minimizes any kind of a-priori constraint to the variables used to retrieve the CBH as well as on the analytical relationship between the variables in the statistical model. This is achieved by training a statistical model based on machine learning. The model is trained using as predictors the observed radiances (GOES-16) and ancillary predictions from the High-Resolution Rapid Refresh (HRRR, [18]) NWP model, and CBH observations from meteorological aerodrome reports (METAR) across the contiguous U.S. (CONUS). The network of METAR stations is dense in the USA and provides higher spatial coverage than other CBH retrievals such us rawinsondes [19] or cloud radars [20,21]. Our results highlight the potential of using geostationary satellites to provide high spatio-temporal monitoring of the CBH field.

The manuscript is organized as follows. Section 2 describes the predictand/predictor datasets and the calibration of the statistical model. Section 3 presents the results, and a discussion is presented in Section 4.

## 2. Materials and Methods

The following subsections describe the observational datasets used to create the predictand/predictors datasets and the training of the machine learning model.

### 2.1. Observational Datasets

To explore the potential of machine learning to retrieve the CBH, we used hourly data over the CONUS for the month of April 2018. The month of April was selected as a transition between the synoptic regimes of the cold winter season and the more warmer regimes of summer in order to maximize the representation of synoptic patterns, and thus cloud features, across the year. The data were interpolated to a grid covering the CONUS at a 9 km grid spacing in order to pair the diverse sets of observations.

**Figure 1.**
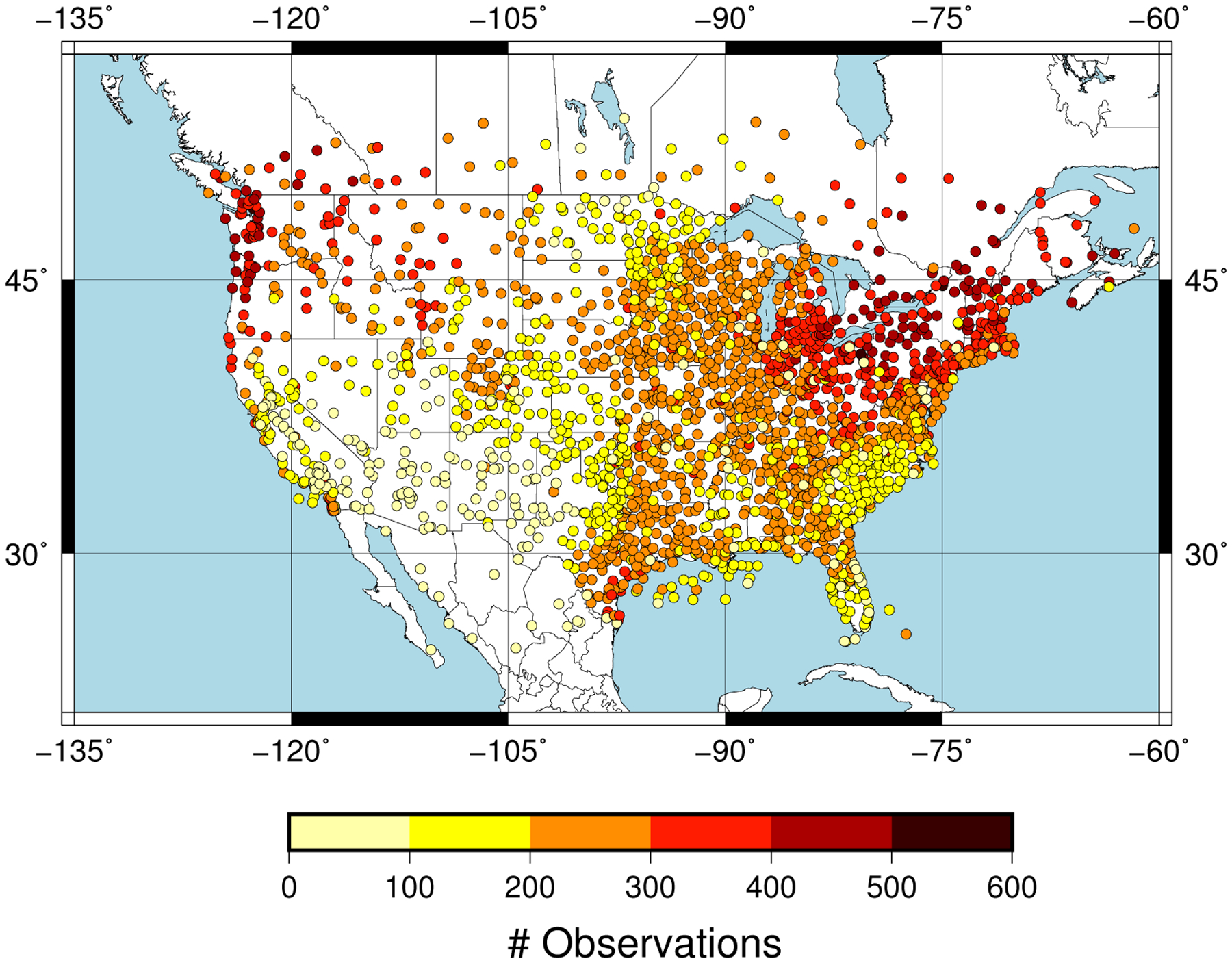
Number of cloud base height (CBH) observations at each meteorological aerodrome report (METAR) station.

**Figure 2.**
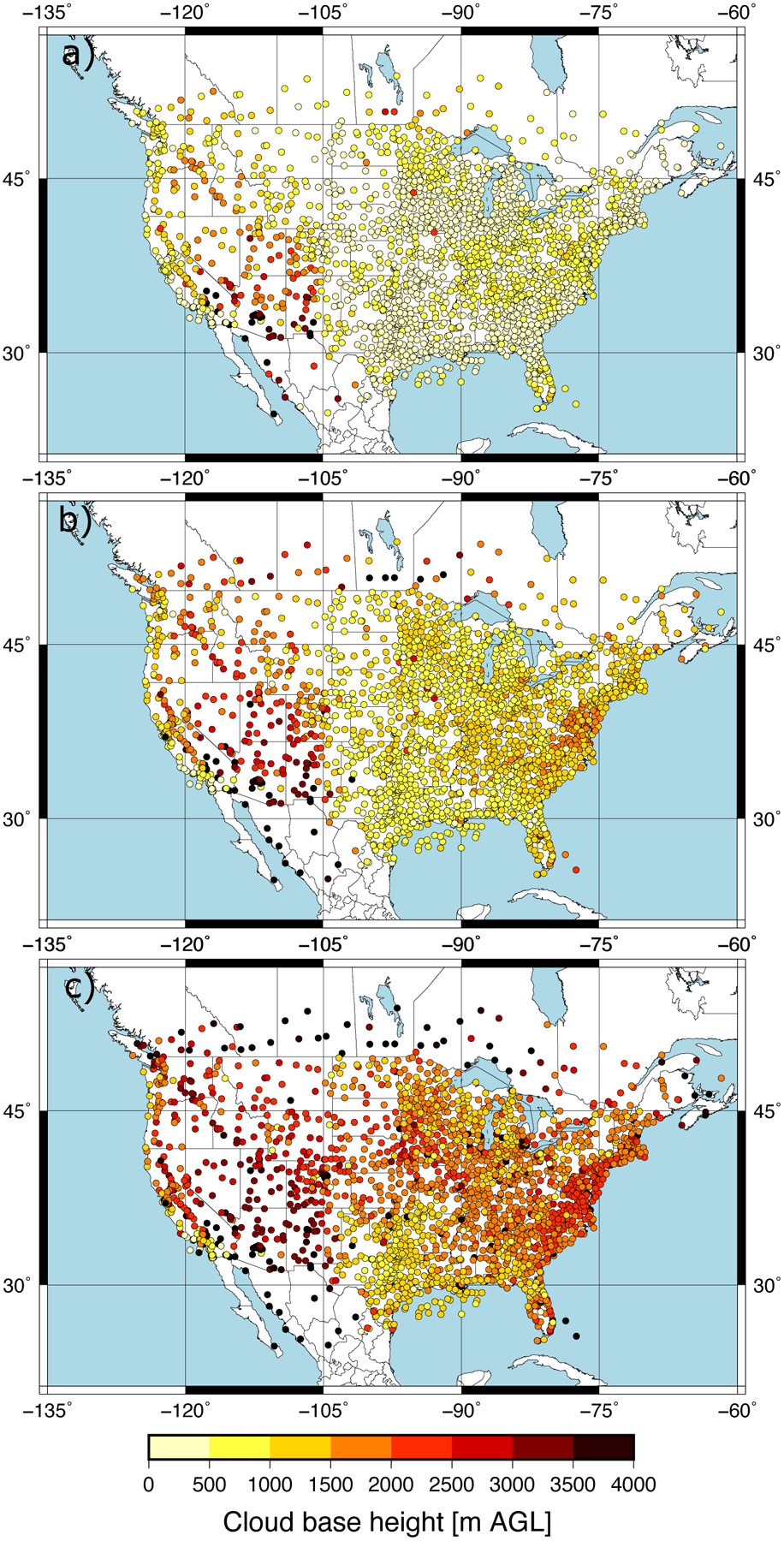
Percentiles of the CBH at each METAR station: 25th percentile (**a**), median (**b**), and 75th percentile (**c**).

The predictand dataset consisted of CBH observations within 10 min to the hour recorded over the CONUS at 2187 METAR stations. We selected the CBH of those observations reporting overcast or broken cloud conditions, since this was consistent with the definition of the ceiling by the aviation community. The METAR sampling of the CBH field over the CONUS is dense. This can be seen in Figure 1, which shows the number of CBH observations available at each site during April 2018. A large number of observations at a given site is a consequence of the greater frequency of cloudy skies, and the spatial pattern is consistent with the spring cloud frequency climatology over the CONUS [22]. The distribution of the CBH was summarized with the 25th, 50th and 75th percentiles (Figure 2). Most of the sites had the 25th percentile within the first 500 m (Figure 2a) with a value of 488 m when all the observations were considered. The 50th percentile (median) showed CBHs between 500 m and 1500 m in the eastern CONUS and the west coast, and between 2000 and 3000 m in western parts of the CONUS (Figure 2b). The median was 1092 m when all the sites were considered. The 75th percentile showed a more homogeneous pattern, with the CBH usually above 1500 m (Figure 2c) and a value of 1981 m when all the observations were combined. The good spatial coverage together with the data availability on an hourly basis (720 h) allowed us to have a sufficient large predictand dataset to explore the performance of the machine learning model.

The predictor dataset consisted of the retrievals from GOES-16 and NWPs. The former were the radiances recorded on the hour with the 16 channels sampled by the ABI instrument in the CONUS scans. The NWP dataset consisted of 1 h predictions from the HRRR model run operationally by the National Center for Environmental Predictions (NCEP). HRRR is based on the Weather Research and Forecasting (WRF) [23] model and issues valid forecasts over the CONUS every hour. The horizontal grid spacing is 3 km. In this study, we used valid 1 h predictions from each hour during the month of April 2018. The 1 h predictions were used to mimic the data availability for CBH monitoring in a potential operational mode. The following HRRR variables were selected as potential predictors for the machine learning model: temperature at 2 m above ground level, ground snow content, ground albedo, skin temperature and relative humidity (RH) at 15 m, 100 m, 250 m, 500 m, 750 m and 1 km above ground level. These variables were complemented with the time, the latitude and longitude and the solar zenith angle to complete the predictor dataset.

**Figure 3.**
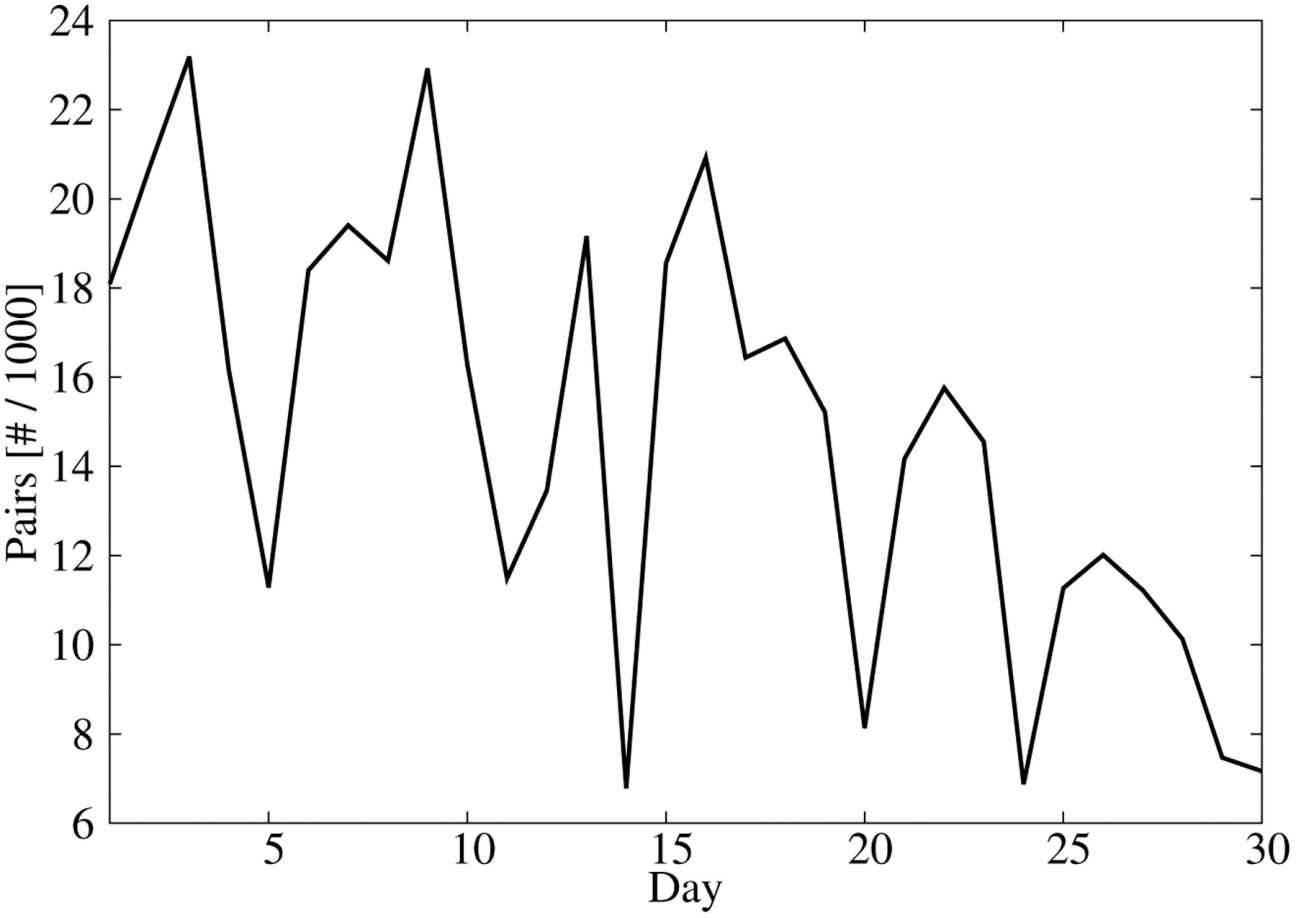
Number of predictand/predictor pairs as a function of the day.

The predictand/predictor dataset was divided into two periods. The first 15 days (360 h) of the month were used to calibrate the machine learning model. This dataset showed a CBH distribution consistent with the distribution during the entire month (Figure 2). The observations for the other 15 days were used to evaluate the performance of the CBH predictions. This resulted in 300,061 pairs of predictand/predictors for the training dataset and 194,322 pairs for the validation dataset. This different number of observations is due to the unavailability of certain GOES-16 radiance scans, the presence of missing values in the radiance scans and the cloud cover variability, which caused some days to have more CBH observations than others. The number of available observations per day was between 7000 and 22,000 (Figure 3).

### 2.2. Machine Learning Model

The machine learning model used in this study was the random forest algorithm [24], which is a machine learning model commonly used in data science applications due to its interpretability and ability to generalize well. Random forests are an ensemble of decision or regression trees and generally avoid overfitting since each tree in the forest is provided a subset of the available predictors and training data. Each tree in the forest is a set of rules, or decisions, that is used in order to minimize the variance or impurity of the response variable, which in this case was the CBH [25]. More details of the random forest machine learning algorithm can be found in [26,27].

The month of April represented a valid test of the methodology due to the diverse set of weather conditions that occur over the CONUS. The CONUS has a great diversity of climates, and the very vast majority of cloud types at different elevations on the surface of the Earth should be present during April. The diversity of cloud frequencies and CBHs over the CONUS has been shown in [22]. Any statistical or machine learning-based methodology will improve with more data to capture the signal within the noise, and this would have been especially true in our case if we were able to obtain a full year of data for training and testing; however, the month of April over the CONUS represented a robust test of the methods.

## 3. Results

### 3.1. Model Calibration

**Figure 4.**
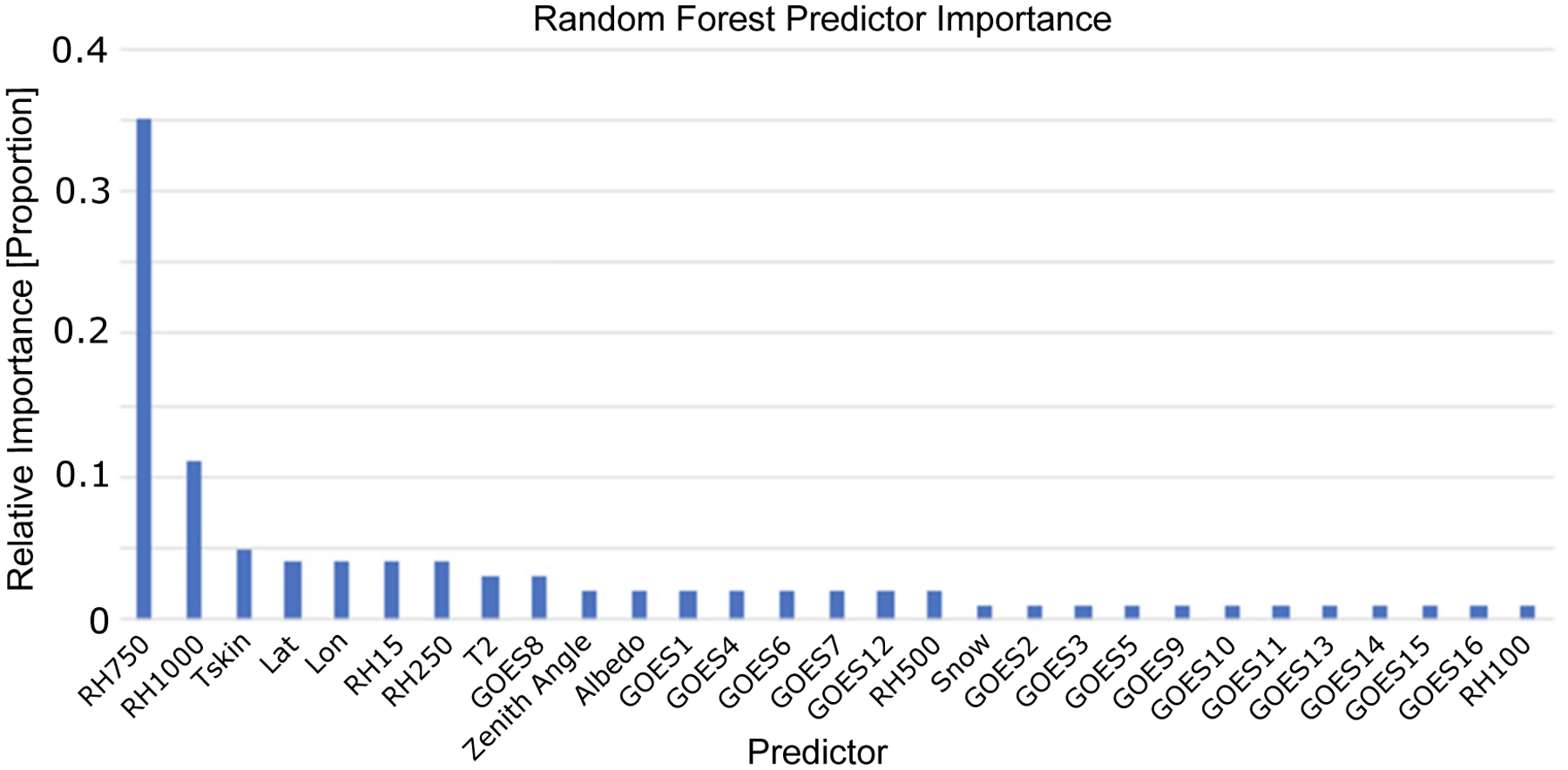
Predictor importance for the random forest model, showing that the majority of the importance originated from the relative humidity (RH) at 750 m and 1000 m.

The random forest was trained on a set of variables from the GOES-16 satellite data and additional derived variables. The variable set included the latitude, longitude, temperature at 2 m and skin temperature, the albedo, the binary classification if snow was present, the solar zenith angle, RH at 15, 250, 500, 750, and 1000 m above ground level and the GOES bands. The model was trained by using the data from 1–15 April, and the model was evaluated on the test dataset of April 16–30. The optimal model hyperparameters were determined using a random 80% split of the training data and determining the model with the optimal mean absolute error (MAE) and correlation coefficient. The optimal configuration of the random forest model was composed of 200 trees, used the mean absolute error (MAE) criteria to measure the quality of a split in the tree and allowed for an automatic estimation of maximum features at each split in Python’s Sci-kit learn package [28]. This final model relied heavily on the RH values at 750 and 1000 m, which was of course physically reasonable given that this represented the typical height levels of clouds, and RH values close to 100% would indicate cloud formation. The predictor importance analysis is shown in Figure 4, where the RH 750 and RH 100 values comprise almost 50% of the weight of the predictors. However, all predictors show at least 1% importance, and this is reasonable given that each variable may add value to clouds at different height levels that occur in different climates with varying frequencies.

While the random forest model showed that most of the value originated from the NWP model, there was still a significant amount of value that originated from the satellite data. In fact, 23% of the predictor importance was from the GOES1–GOES16 variables. More significantly, the physical representation of these variables is important to keep in mind when determining which variables to keep. The importance of the NWP RH levels was representative of the fact that these were the heights at which most clouds are present in the atmosphere, but the GOES variables may have added skill in cloud types at different levels such as high cirrus or low stratus, and therefore all variables were retained in the final predictor dataset.

### 3.2. Model Performance

**Figure 5.**
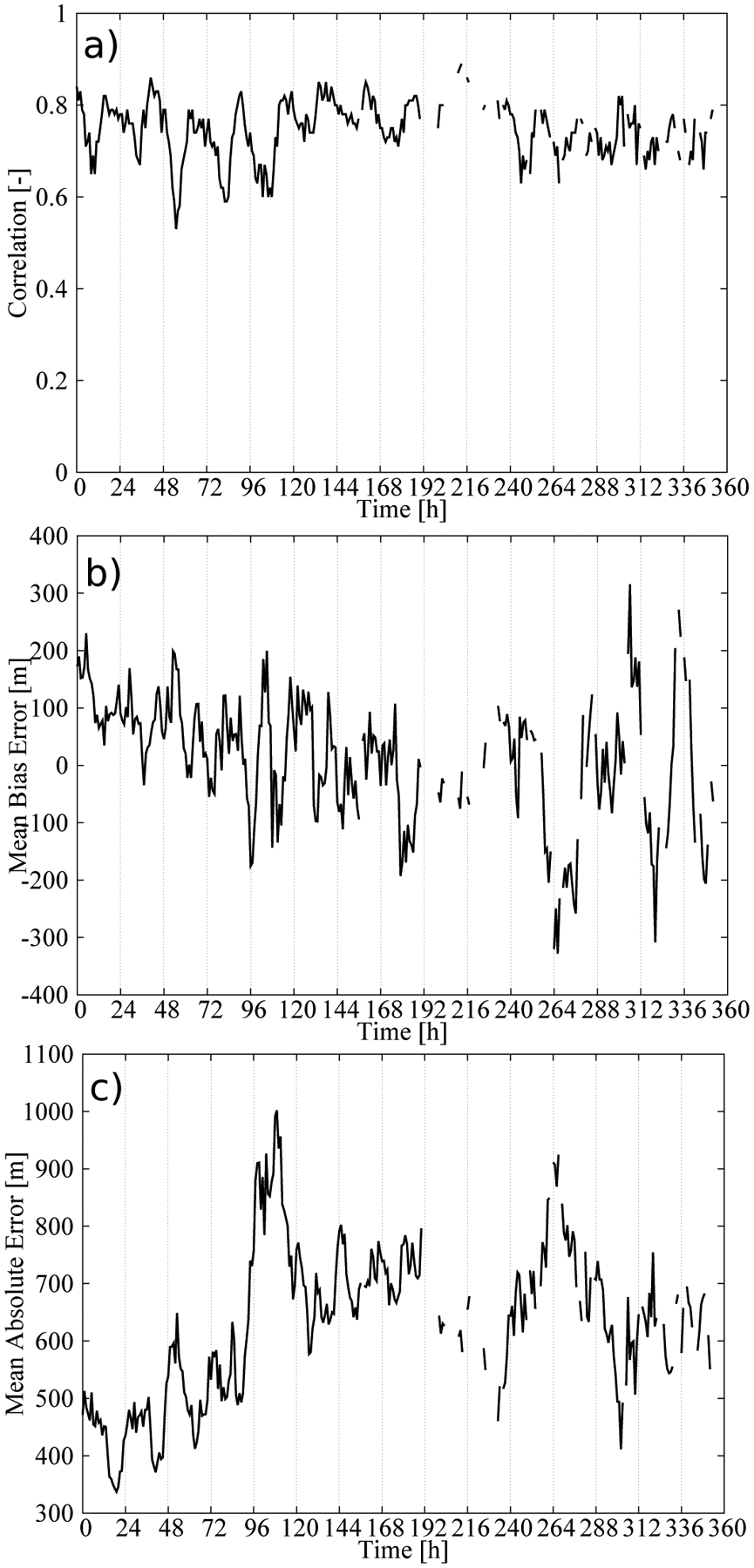
Hourly statistics as a result of comparing the observed and predicted CBH using the validation dataset: (**a**) Spatial correlation, (**b**) mean bias error (MBE) and (**c**) mean average error (MAE).

Figure 5 shows the hourly time series of three statistics summarizing the performance of our statistical model in estimating the CBH field: the spatial correlation, the mean bias error (MBE) and the MAE. At each hour, the statistics were calculated by comparing the estimations from the statistical model against the METAR observations reporting the CBH. The process was repeated for 360 h of the validation dataset to calculate the time series of the statistics. The time series of the correlation (Figure 5a) consistently showed high values. The smallest values were around 0.6, but certain hours showed values above 0.8, with a correlation of 0.77 when all pairs of observations and estimations were considered. This strong positive correlation indicates that the statistical model captures the spatial variability of the CBH field.

**Figure 6.**
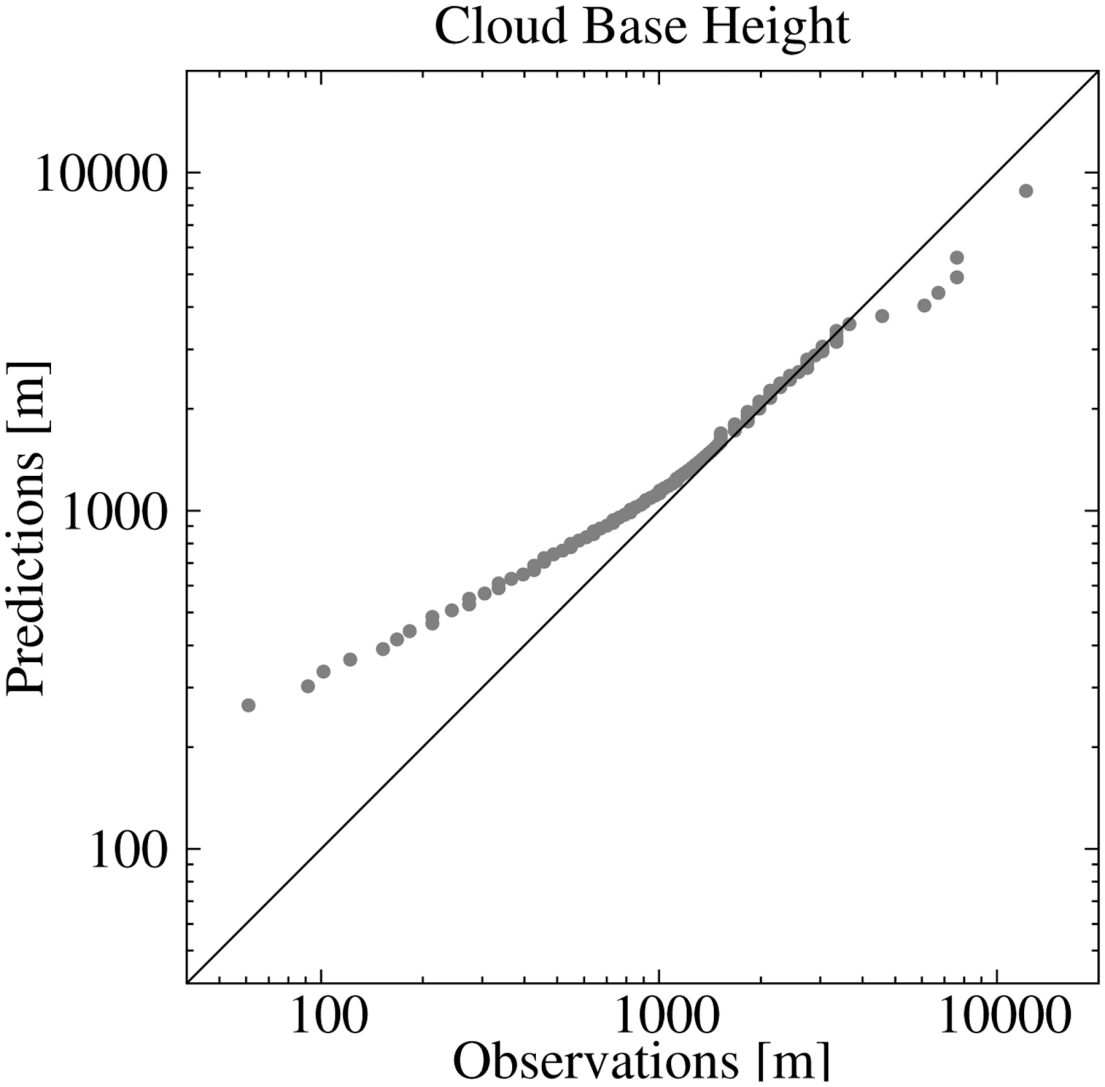
Percentile–percentile comparison of the observed and estimated CBH using all the observations/estimations from the validation dataset.

The time series of the MBE (Figure 5b) did not show a systematic behavior, and it was only 22 m when all observations/estimations pairs were considered. This is a desirable characteristic of the statistical model. However, examining the percentile–percentile plot, or qq-plot, of the estimated and observed CBHs reveals conditional biases in the model (Figure 6). Note the use of logarithmic scales to emphasize the differences. The tendency of the statistical model to overestimate (underestimate) the lowest (highest) CBHs is evident; CBH estimations lower (higher) than 1000 m (4000 m) were underestimated (overestimated). More specifically, the MBE for the CBH below the 25th percentile (488 m) was 446 m, that for CBH between the 25th percentile and the 75th percentile (1981 m) was 214 m, and that for CBH above the 75th percentile was −718 m. This can be understood as a result of averaging the estimations from several trees. In our model, we used 200 trees, which means that the final estimation was the average of 200 estimations—one from each tree. This tended to smooth the lowest (highest) CBH estimations. The length of the training dataset may also have contributed to this behavior, particularly for the higher CBHs, since values higher than 4000 m were less frequent (approximately 6% of the dataset).

**Figure 7.**
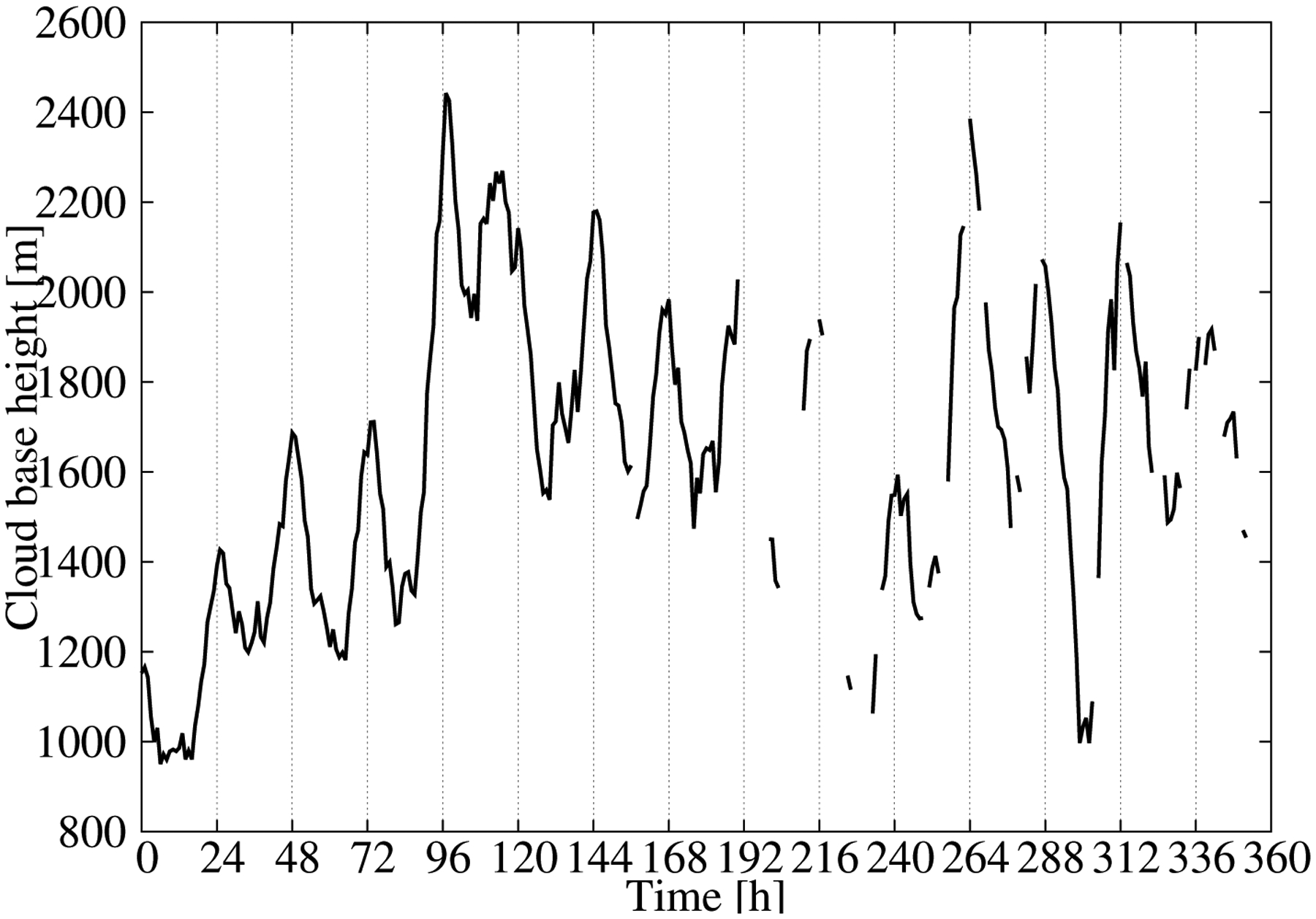
Observed mean CBH for each hour of the validation dataset.

**Figure 8.**
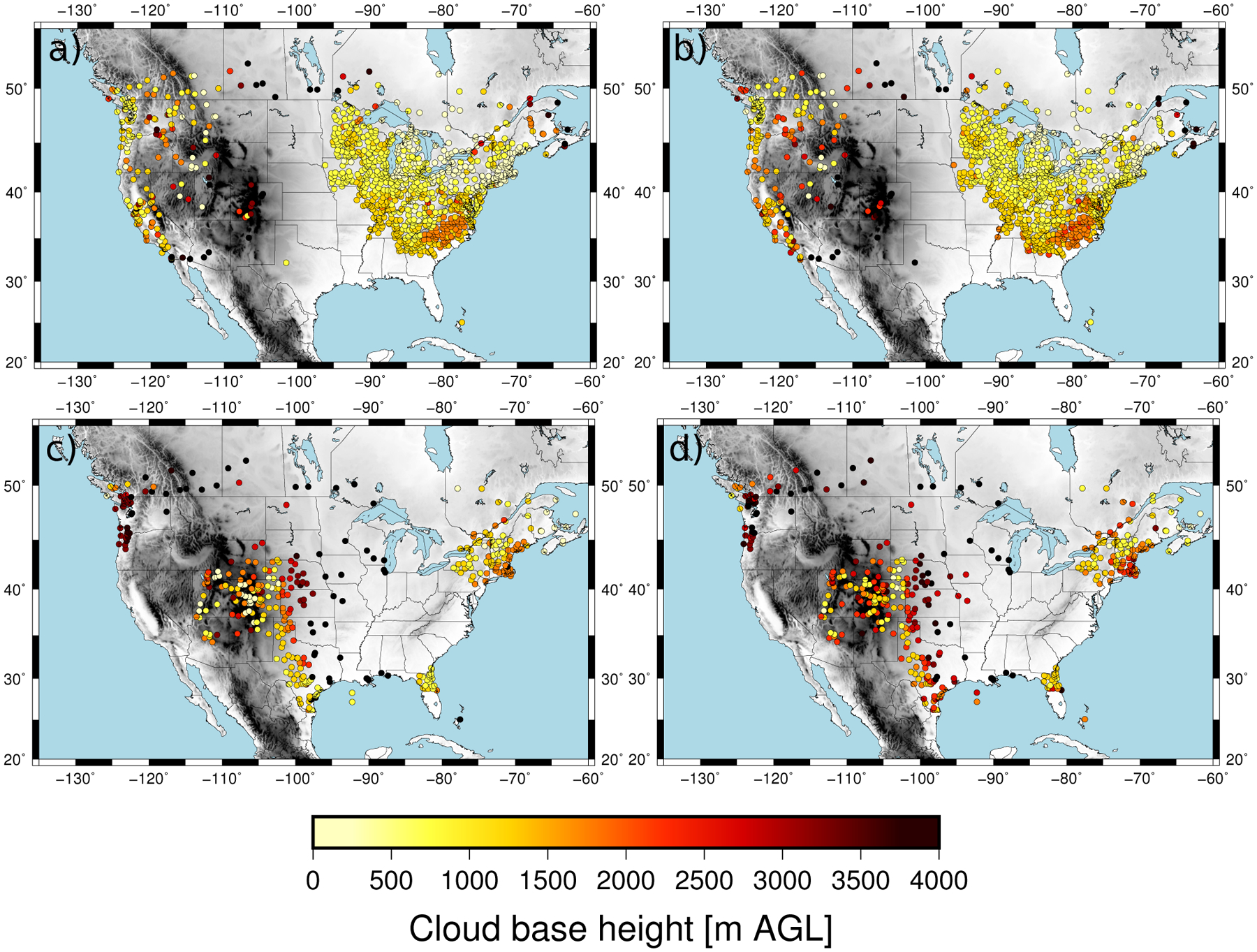
Observed (**left**) and estimated (**right**) CBH for April 16 at 18 UTC (**top**) and for April 20 at 18 UTC (**bottom**).

The time series of the MAE showed values around 450 m during the first few days of the validation dataset and around 700 m for the rest of the time (Figure 5c). As a result, the MAE, considering all pairs of observations/estimations, was 602 m. The error variability can be understood in terms of the mean CBH of the field (Figure 7). The mean CBH time series showed a positive correlation with the MAE time series (correlation = 0.82) revealing that higher (lower) CBHs were associated with higher (lower) errors in the estimations. The error as a function of the CBH was quantified with the MAE for the CBH subsamples below the 25th percentile, between the 25th and 75th percentiles and above the 75th percentile, which were 452 m, 406, and 1115 m, respectively. This behavior is further illustrated in Figure 8, showing the observed and predicted CBH for cases with high (April 20 at 18 UTC) and low (April 16 at 18 UTC) MAE. In both cases, the estimations represent the spatial variability of the CBH well. On April 16, there was a large system on the eastern part of the CONUS with a low CBH (lower than 1000 m), and less structured cloud systems with bases generally lower than 3000 m in the West (the mean CBH was 1077 m). On the contrary, on April 20, there were several cloud structures with CBHs higher than 4000 m in the northwest and in central parts of the CONUS (mean CBH is 2242 m). As a result of the positive correlation, the MAE was higher for the April 20 case (815 m) than for the April 16 case (343 m).

**Figure 9.**
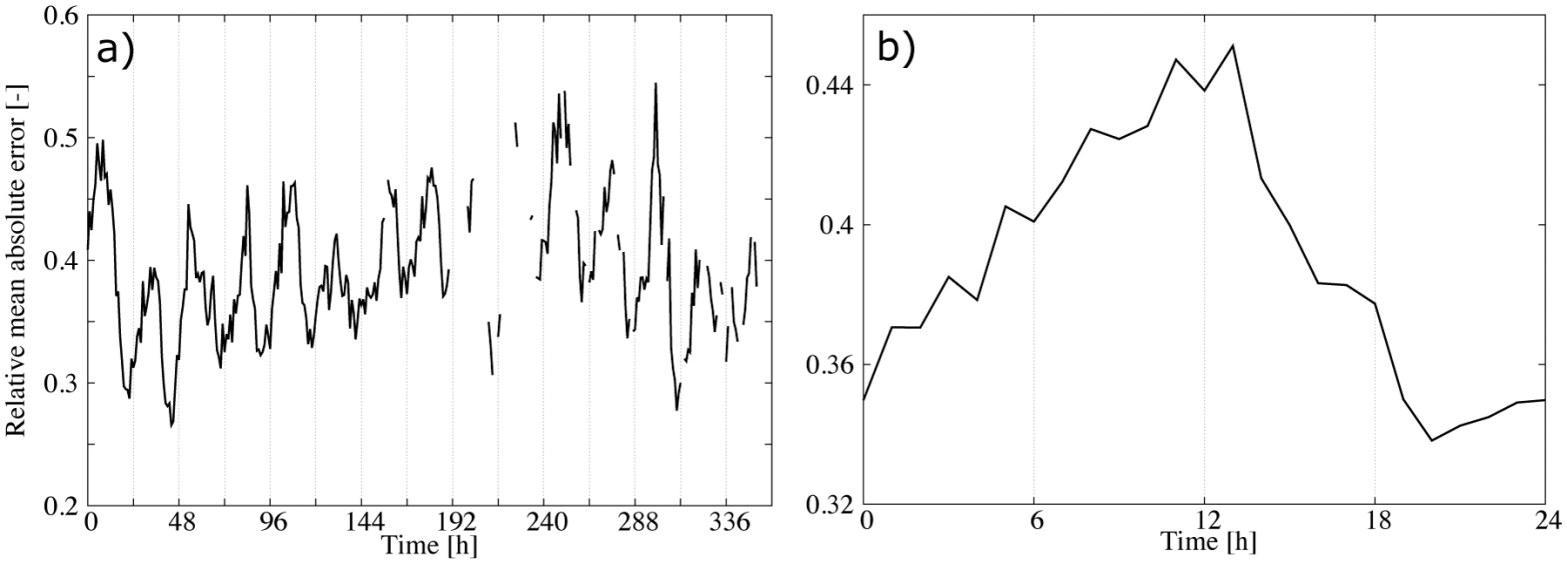
(**a**) Hourly MAE (Figure 5c) normalized by the mean CBH (Figure 7), and (**b**) its dirunal average.

The correlation between the MAE and the mean CBH suggests that the performance of the statistical model can be summarized by normalizing the error by the mean CBH. Figure 9a shows the hourly MAE (Figure 5c) normalized by the mean CBH (Figure 7). As expected, the time series of the normalized MAE did not show important trends or abrupt changes in performance from day to day. The relative error was around 35–40% with a diurnal variability of about 10% (Figure 9b). This result summarizes the performance of the statistical model in retrieving the CBH well. It is relevant that the mean of the predictions was nearly unbiased (Figure 5b), which indicated that we could approximate the normalized MAE using the mean of the predictions. This allowed us to provide both estimations of the CBH and its error.

## 4. Discussion and Conclusions

The benefits of our approach stand out when comparing its performance with other CBH retrievals suffering from an accumulation of errors. For example, the performance of the S-NPP VIIRS was summarized in [17]. The evaluation used CBH retrievals from CloudSat and avoided cases with the CBH or CTH identified within the first km above ground level or cases with precipitation, since the CloudSat retrievals are less reliable under these conditions. A total of 350,521 matchup pairs were identified. The correlation, MBE and root mean square error (RMSE) were 0.185, −700 m and 3500 m, respectively. The performance was greatly improved if matchup pairs with a CTH larger than 2 km were excluded (CTH larger than specifications). This reduced the size of the dataset to 151,274 matchup pairs. Thus, 57% of the CBH retrievals were affected by large errors in the CTH retrievals. The correlation, MBE and RMSE for this reduced set of CBH retrievals were 0.569, −400 m and 2300 m. There was certainly an improvement with a large increase in the correlation and reduction of the MBE and MAE. Our statistical approach, which is independent of the CTH retrieval and thus provides retrievals under all sky conditions, showed better scores: the correlation, MBE and RMSE were 0.77, 22 m and 983 m, respectively. One difference between these evaluation approaches is that the performance of the S-NPP VIIRS was quantified using observations over the entire globe, whereas in this study we focused on the area over the CONUS. Furthermore, many METAR stations do not detect clouds higher than 3660 m [29], and higher CBHs have been shown to be more difficult to predict with our model.

An improved version of the S-NPP VIIRS algorithm [5], which is in the process of being implemented in the NOAA-20 VIIRS, has reduced the dependence of upstream retrievals adding statistical modeling. The algorithm is still dependent on adequate CTH retrievals. An evaluation using 95,145 matchup pairs improved the correlation, MBE and MAE of the S-NPP algorithm. The correlation, MBE and MAE were improved from an original set of 0.452, 700 m and 2700 m to the set of 0.791, 300 m and 1700 m [5]. This improvement was a result of reducing the dependency in upstream retrievals on introducing linear regressions. The statistics do not include the influence of erroneous CTH retrievals, since cases with CTH retrievals with an error larger than 2 km were not included in the calculations. As a result, the errors are expected to increase for CBH under all-sky conditions. Our statistical algorithm showed superior statistics for all-sky conditions. Again, the improved version of the S-NPP algorithm was evaluated using global observations, whereas our statistical model was evaluated with data over the CONUS.

An important conclusion of this work is the importance of having RH estimations from NWP models in the retrieval of the CBH. This becomes evident in the analysis of the predictor importance (Figure 4) that shows RH at 750 and 1000 m above ground level as the most important predictors. The NWP model does not necessarily need to reproduce all the clouds for the statistical model to provide estimations in all instances; indeed, 23% of the clouds in the validation dataset were not captured by the NWP model. For the detected clouds, the statistical model improved the NWP statistics: the MAE decreased from 682 m to 505 m (22% improvement), the MBE was reduced from −356 m to 50 m (86% improvement) and the correlation increased from 0.68 to 0.76 (12% improvement). Obviously, better CBH estimations will be obtained with more realistic RH predictions. RH is routinely sampled by upper-air observations from rawinsondes launched twice a day. The NWP assimilate these observations, which should prevent the RH from drifting too far off from the correct value. Thus, it is a reasonably well-modeled variable that considerably increases the predictability of CBH. Using RH profiles to estimate the CBH is therefore analogous to having temperature profiles to estimate the CTH from passive sensors. In this work, we used the RH humidity at low atmospheric levels, but including RH at higher levels should help to increase the predictability of CBH.

The errors in the methodology that we propose would likely be further reduced by using a multi-year training period, including data from other months and seasons and using a comprehensive selection of predictors from the HRRR model. This would allow for a more generalizable and robust model. However, our results are sufficient to support our working hypothesis that avoiding errors in upstream retrievals is desirable to improve the performance of CBH retrievals.

## Supplementary Material

dataset_training

dataset_validation
